# Oxidative Stress of Office Workers Relevant to Tobacco Smoking and Inner Air Quality

**DOI:** 10.3390/ijerph110605586

**Published:** 2014-05-26

**Authors:** Chung-Yen Lu, Yee-Chung Ma, Pei-Chun Chen, Chin-Ching Wu, Yi-Chun Chen

**Affiliations:** 1 School of Post-Baccalaureate Chinese Medicine, China Medical University, Taichung 404, Taiwan; E-Mail: u100030082@cmu.edu.tw; 2 Research Center for Traditional Chinese Medicine, China Medical University Hospital, Taichung 404, Taiwan; 3 Institute of Environmental Health, National Taiwan University, Taipei 100, Taiwan; E-Mail: ycma@ntu.edu.tw; 4 Institute of Epidemiology and Preventive Medicine, National Taiwan University, Taipei 100, Taiwan; E-Mail: peichunchen@ntu.edu.tw; 5 Department of Public Health, China Medical University, Taichung 404, Taiwan; E-Mail: wucc@mail.cmu.edu.tw; 6 Department of Health Management, I-Shou University, Kaohsiung 824, Taiwan

**Keywords:** carbon dioxide, cotinine, 8-hydroxydeoxyguanosine, office worker, tobacco smoking

## Abstract

Studies have used 8-hydroxydeoxyguanosine (8-OHdG) as a biomarker to detect systemic oxidative DNA damage associated with oxidative stress. However, studies on the association between exposure to tobacco smoking and urinary 8-OHdgG give inconsistent results. Limited studies have estimated the oxidative stress among office workers. This study assessed the association between urinary 8-OHdG and cotinine for office workers. Workers (389) including smokers, ex-smokers and non-smokers from 87 offices at high-rise buildings in Taipei participated in this study with informed consent. Each participant completed a questionnaire and provided a spot urine specimen at the end of work day for measuring urinary 8-OHdG and cotinine. The carbon dioxide (CO_2_) levels in workers’ offices were also measured. The questionnaire reported socio-demographic characteristics, life styles and allergic history. The urinary 8-OHdG level increased with the cotinine level among participants (Spearmans’ rho = 0.543, *p* < 0.001). The mean of urinary 8-OHdG and cotinine was 5.81 ± 3.53 μg/g creatinine and 3.76 ± 4.06 μg/g creatinine, respectively. Comparing with non-smokers, the adjusted odds ratio (OR) of having urinary 8-OHdG greater than the median level of 4.99 μg/g creatinine was 5.30 (95% confidence intervals (CI) = 1.30–21.5) for current smokers and 0.91 (95% CI = 0.34–2.43) for former smokers. We also found workers exposed to 1,000 ppm of CO_2_ at offices had an adjusted OR of 4.28 (95% CI = 1.12–16.4) to have urinary 8-OHdG greater than 4.99 μg/g creatinine, compared to those exposed to indoor CO_2_ under 600 ppm. In conclusion, urinary 8-OHdG could represent a suitable marker for measuring smoking and CO_2_ exposure for office workers.

## 1. Introduction

Oxidative stress has been associated with the risk of cancer, degenerative diseases, such as heart disease and Alzheimer’s disease, and chronic inflammation [1,2,3]. Reactive oxygen species (ROS) such as superoxide radicals, singlet oxygen, hydrogen peroxide, and hydroxyl radicals may lead to oxidative damage. ROS can be generated endogenously from physiologic process and exogenously because of exposing to ultraviolet light, air pollution as well as tobacco smoking.

More than 4,800 chemicals have been determined in tobacco smoke, including various free radicals or ROS, which can cause damage in cellular membrane lipids, proteins, enzymes and DNA [4,5]. Cigarette and tobacco smoking-induced oxidative damage is highly associated with chronic disorders, including atherosclerosis, cancers, and chronic obstructive pulmonary disease [6,7] and male infertility [8].

8-Hydroxydeoxyguanosine (8-OHdG), generated by the interaction of hydroxyl radical with guanine [9], has been associated with lung cancer [9,10,11] and neurodegenerative diseases [12]. Several studies have used 8-OHdG as a biomarker to detect systemic oxidative DNA damage [9,13] to estimate oxidative stress linking occupational and environmental exposures [14,15,16,17,18,19]. Wang *et al.* [19] used 8-OHdG as a biomarker to evaluate chromate producing facilities and found chromate decreased serum folate and increased the risk of DNA damages in workers. Han *et al.* [17] found that urinary 8-OHdG levels were higher in bus drivers than in office workers. However, studies in the association between 8-OHdG and exposure to tobacco smoke have resulted in inconsistent findings. Harman *et al.* [20] reported that the urinary 8-OHdG levels were not associated with smoking status among healthy community participants. A survey among women working at laundry shops also found no association between urinary 8-OHdG and smoking status [14]. On the other hand, Asami *et al.* [21] found the level of 8-OHdG in lung tissues was 1.43-fold higher in smokers than in non-smokers. Campose *et al.* [22] have also found recently higher urinary 8-OHdG levels in healthy cigarette smokers than in non-smokers (10.7 ng/mg creatinine *vs.* 8.3 ng/mg creatinine). Increased excretion rate of urinary 8-OHdG also has been associated with cigarette smoking, even in lung cancer patients [23].

Information on smoking in the above referred studies was mainly collected with questionnaires, which may have recall bias leading to inconsistent findings. Urinary cotinine, one of metabolites of nicotine, is a good and specific biomarker of tobacco smoking exposure [24]. Using a biomarker associated smoking would increase the measured precision for the association between 8-OHdG and smoking. Besides, the relationship between urinary cotinine and 8-OHdG may reveal the body systemic oxidative stress from tobacco smoking. In high-rise buildings, office workers may expose to indoor air pollutants such as carbon dioxide, volatile organic compounds and tobacco smoking associated with the air ventilation systems [25]. Few studies have focused on office workers to demonstrate whether smoking is the major source of indoor air pollution for employees in high-rise buildings. However, other pollutants in the office air may also contribute to the oxidative damage. Carbon dioxide (CO_2_) has been used as an indicator of indoor air quality [26]. Therefore, this study investigated the association between urinary 8-OHdG and cotinine among office workers. We also evaluated the correlation between urinary 8-OHdG and indoor CO_2_ for office workers.

## 2. Methods

### 2.1. Study Subjects and Data Collection

Employees working with 16 government agencies and commercial organizations located in high-rise building offices in Taipei City, Taiwan were invited to participate in this study for investigating the association between sick building syndrome and indoor air pollutants. The details of this study have been reported in previous studies [25]. An invitation letter explaining the study was delivered to potential participants at 87 offices. With informed consent, 398 workers (response rate 61.7%) completed the self-reported questionnaires and provided spot urine samples for measuring 8-OHdG and cotinine levels. The questionnaire provided the information on sex, age, education, smoking history, allergic history, office characteristics, using cleaning chemicals and complaints of sick building syndromes. The CO_2_ levels in workers’ offices were also measured. This study was approved by an institutional review committee.

### 2.2. Urine Sample Analysis

Each participant provided the spot urine sample at the end of the workday. An investigator collected specimens of urine, transported them at 4 °C in a cold box and stored them at −80 °C in a freezer until analysis. The 8-OHdG levels in all urine samples were determined within 2 months. Urine samples were centrifuged at 3,000 rpm for 10 min after being thawed to remove particles in samples.

Urinary cotinine was measured with rapid analysis using a direct barbituric acid assay based on the König reaction [27,28,29]. At room temperature, we took 400 μL of urinary sample and prepared standard solutions with 5, 10, 50, 100, 150, 200, and 250 μmol/L cotinine for analysis, with the *r*^2^ values of the calibration curves ranged between 0.99 and 0.999. Cotinine concentration was measured by comparing the absorbance value at the wave length of 550 nm with a detection limit of 2.37 μg/L. The OXIS Research enzyme-linked immunosorbent assay (ELISA) kit (Japan Institute for the Control of Aging, Shizuoka, Japan) measured 8-OHdG in the urine. Fifty μL of 8-OHdG monoclonal antibody was added to 50 μL of urine sample or standard solution onto a micro-titer plate with pre-coated 8-OHdG for assay at room temperature [30]. Urinary 8-OHdG levels were determined against 0.5, 2, 8, 20, 80, and 200 ng/mL 8-OHdG with a reagent blank, with the *r*^2^ values of the calibration curves >0.980 and the method detection limit of 0.26 μg/L. The urinary 8-OHdG levels were adjusted with urinary creatinine and expressed as μg/g creatinine.

Urinary creatinine levels were measured using an automatic analyzer (Hitachi 7250, Tokyo, Japan) with the Jaffe colorimeter reaction [31]. The urinary concentrations of both 8-OHdG and cotinine were adjusted with creatinine levels.

### 2.3. Statistical Analysis

Nine participants who provided no urinary specimens were excluded in the data analyses. Data analyses first calculated means and standard deviations for creatinine adjusted cotinine and 8-OHdG in the urine samples by smoking status for females and males. Kruskal-Wallis test was used to examine differences. The correlations between urinary cotinine and 8-OHdG were plotted by smoking status and examined by Spearmans’ rho. We further used the median level of 8-OHdG (4.99 μg/g creatinine) among all participants as the cut-off to express the elevated oxidative stress because of skewed distribution. The concentrations of urinary cotinine were stratified into quartile levels, *i.e.*, <1.55, 1.56–2.32, 2.33–3.98, >3.98 μg/g creatinine to express the exposure of smoking for participants. Considering the other indoor air pollutants might be associated with levels of urinary 8-OHdG, the indoor concentration of carbon dioxide (CO_2_) (<600, 600–1,000, >1,000 ppm) and volatile organic compounds (VOCs) (500, >500 ppb) were included in this study as covariates as well. The logistic regression analysis was used to estimate the odds ratios (OR) and 95% confidence intervals (CI) of elevated urinary 8-OHdG in association with smoking status and urine cotinine levels controlling for age, gender, indoor temperature and relative humidity as well as the classification of indoor concentrations of carbon dioxide and volatile organic compounds. Data analyses were performed using the statistical package software of SAS 8.1 (SAS Institute Inc., Cary, NC, USA) and Excel, and α value was taken as 0.05.

## 3. Results

### 3.1. urinary Cotinine and 8-OHdG of All Subjects

**Figure 1:**
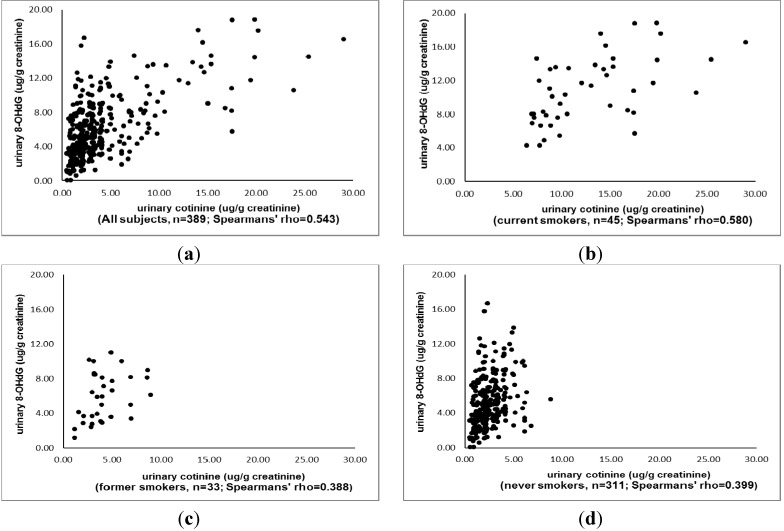
The scatter plots and Spearmans’ rho between urinary cotinine and 8-OHdG by smoking status. (**a**) for all subjects; (**b**) for current smokers; (**c**) for former smokers and (**d**) for never smokers.

Among the 389 office workers who participated in this study with complete data, 77.1% were females, 30.8% were older than 40 years and 45.8% had worked more than 4 years (data not shown). Table 1 shows distributions of urinary cotinine and 8-OHdG among participants by smoking status and gender. Among all participants, the mean urinary cotinine and 8-OHdG were 3.76 ± 4.06 μg/g creatinine and 5.81 ± 3.53 μg/g creatinine, respectively. The mean urinary cotinine was higher in men than in women (5.76 ± 5.11 *vs.* 3.17 ± 3.49 μg/g creatinine). The corresponding levels of mean urinary 8-OHdG were 6.88 ± 3.71 and 5.51 ± 3.39 μg/g creatinine. Urinary cotinine and 8-OHdG levels of office workers were associated with their smoking status. The concentrations of both urinary cotinine and 8-OHdG were much higher in current smokers than in former smokers and in non-smokers (*p* < 0.01). Figure 1 shows that the urinary level of 8-OHdG increased as the urinary level of cotinine increased, with Spearman’s rho of 0.543 (*p* < 0.001) for all participants and 0.580 (*p* < 0.001) for current smokers. The correlations between urinary cotinine and 8-OHdG for former smokers and non-smokers declined.

**Table 1:** Means and standard deviations of urinary cotinine and 8-hydroxydeoxyguanosine (8-OHdG) levels measured for office employees by smoking status and gender.

Smoking status		Females			Males				All				
	*N*	Means (SD ^a^)	*N*		Means (SD ^a^)		*p*-value		*N*		Means (SD ^a^)		
Cotinine (μg/g creatinine)													
Non-smoker	270	2.36 (1.29)	41		2.57 (1.60)		0.346		311		2.39 (1.33)		
Ex-smoker	10	3.90 (1.37)	23		4.34 (2.34)		0.586		33		4.21 (2.08)		
Smoker	20	13.7 (6.38)	25		12.3 (4.75)		0.397		45		12.9 (5.51)		
Total	300	3.17 (3.49)	89		5.76 (5.11)		<0.001		389		3.76 (4.06)		
*p*-value		<0.001			<0.001						<0.001		
8-OHdG (μg/g creatinine)													
Non-smoker	270	5.07 (2.88)	41		5.08 (2.56)		0.963		311		5.08 (2.84)		
Ex-smoker	10	5.65 (2.85)	23		6.05 (2.76)		0.705		33		5.93 (2.75)		
Smoker	20	11.3 (3.39)	25		10.6 (3.49)		0.534		45		10.9 (3.96)		
Total	300	5.51 (3.39)	89		6.88 (3.71)		0.001		389		5.81 (3.53)		
*p*-value		<0.001			<0.001						<0.001		

Note: ^a^ SD = standard deviation.

### 3.2. The Association between Urinary 8-Ohdg and Cotinine

Table 2 displays factors that may predict the urine 8-OHdG at a level higher than the median (4.99 μg/g creatinine) of all participants, including sex, age, smoking status, urine cotinine level, indoor temperature and relative humidity, and the indoor concentration of carbon dioxide and volatile organic compounds. 

**Table 2:** Odds ratio of having urine 8-hydroxydeoxyguanosine (8-OHdG) greater than median, 4.99 μg/g creatinine, by age, gender, temperature, relative humidity, the concentration of indoor carbon dioxide and volatile organic compounds, smoking status and urinary cotinine, (*N* = 389).

Variables (*n*)	Odds Ratio (95% confidence interval)
Age, years old	
<29 (101)	1.0
30–39 (168)	0.76 (0.42, 1.38)
40–49 (62)	0.85 (0.40, 1.83)
50+ (58)	1.21 (0.54, 2.68)
Gender	
Female (300)	1.0
Male (89)	0.96 (0.47, 1.93)
Indoor temperature, °C	
≤24 (239)	1.0
>24 (150)	0.51 (0.24, 1.09)
Relative humidity, %	
≤60 (322)	1.0
>60 (67)	0.57 (0.24, 1.33)
CO_2_, ppm	
<600 (47)	1.0
600–1000 (193)	3.38 (1.24, 9.23)
>1000 (149)	4.28 (1.12, 16.4)
VOCs, ppb	
≤500 (294)	1.0
>500 (95)	1.80 (0.82, 3.95)
Smoking status	
Never (311)	1.0
Former (33)	0.91 (0.34, 2.43)
Current (45)	5.30 (1.30, 21.5)
Urine cotinine level, μg/g creatinine	
≤1.55 (98)	1.0
1.56–2.32 (95)	0.95 (0.48, 1.89)
2.33–3.98 (96)	3.30 (1.67, 6.54)
>3.98 (100)	6.66 (2.80, 15.8)

Age, sex, indoor temperature, relative humidity and VOCs were not associated with elevating the level of urinary 8-OHdG. The concentration level of indoor CO_2_ was significantly associated with the elevated urinary 8-OHdG. The adjusted OR was 4.28 (95% CI = 1.12–16.4) for office workers exposed to the concentration of indoor CO_2_ more than 1000 ppm compared with those exposed to indoor CO_2_ under 600 ppm. Compared with non-smokers, the adjusted OR was 5.30 (95% CI = 1.30–21.5) for current smokers, but was 0.91 (95% CI = 0.34–2.43) for former smokers. The OR of having elevated urinary 8-OHdG increased with the urinary cotinine level. Compared to workers with urine cotinine levels under 1.55 μg/g creatinine, the adjusted OR was 3.30 (95% CI = 1.67–6.54) for those with cotinine levels at 2.33–3.98 μg/g creatinine. The OR increased to 6.66 (95% CI = 2.80–15.8) for those with the urine cotinine level of above 3.98 μg/g creatinine.

## 4. Discussion

To the best of our knowledge, limited studies have reported the relationship between oxidative stress and smoking exposure for white-collar employees [17]. In this study, we found a strong correlation between urinary 8-OHdG levels and cotinine levels among employees at smoking prohibited offices (Spearmans’ rho = 0.543, *p* < 0.001) and significantly associated with smoking status and indoor CO_2_. The mean urinary 8-OHdG of current smokers is 2.15-fold and 1.17-fold higher than means of non-smokers and former smokers, respectively. Compared with non-smokers, the OR of elevated 8-OHdG levels for former smoker was 0.91 (95% CI = 0.34–2.43) but the OR increased to 5.30 (95% CI = 1.30–21.5) for current smokers in the multivariate logistic regression analysis. The findings reflect that non-smokers and ex-smokers have very low nicotine exposure in the offices. The elevated 8-OHdG levels in self-reported smokers reflect that smoking leads the extra DNA damage. These results are consistent with previous studies [22,23,32]. Campos *et al.* [22] found that oxidative stress from cigarette did not appear in former smokers. Morita *et al.* [32] have shown that quitting smoking for two weeks may quickly reduce the level of urinary 8-OHdG and improve the platelet aggregation.

A Japanese study found in patients with lung cancer that the excretion rate of urinary 8-OHdG is associated with the duration of smoking cessation [23]. Current smokers had the highest excretion rate of urinary 8-OHdG within two weeks, compared with former smokers and never smokers [23]. Quitting smoking may quickly reduce the oxidative stress from tobacco smoking.

Studying oxidative stress associated with tobacco smoking, we should also consider exposure from environmental tobacco smoking. The cotinine detected in low level may be due to environmental tobacco smoking exposure in our study. The smoking free worksite campaign has been promoted in Taiwan [33]. Therefore, the office workers in our study might have occasional exposure to environmental tobacco smoke at other locations instead of workplaces. The mean urinary cotinine is higher in former smokers than in never smokers could be due to higher environmental smoking exposure. Matt *et al*. [34] found higher concentration of nicotine in the air of smokers’ houses, even after an effort of clean, paint and vacant for 2 months, than in non-smokers’ home.

This study found that the elevated level of urinary 8-OHdG among office workers was also positively associated with indoor carbon dioxide concentrations in offices in addition to the tobacco smoke. We found that workers at offices with high CO_2_ had an odds ratio of 4.28 to have a high urinary 8-OHdG, compared with those at offices with low CO_2_. CO_2_ levels also reflect the indoor air quality [26]. Poor ventilation may increase not only CO_2_ levels in offices of high-rise buildings but also other pollutants with the oxidative stress effect. Therefore, office workers working in poorly ventilated environment are at a higher risk to have elevated 8-OHdG.

The average urinary 8-OHdG measured for male office workers in our study was 6.88 ± 3.71 μg/g creatinine, which is lower than the level for male office workers at bus stations with a mean of 7.3 ± 5.4 μg/g creatinine [17], men working at a chromate manufacturing plant (7.96 ± 7.71 μg/g creatinine) [19], taxi drivers (13.4 ± 4.7 μg/g creatinine) [35], or bus drivers (9.5 ± 5.7 μg/g creatinine) [17]. Han *et al.* [17] found that exposure to the traffic exhaust increased the urinary 8-OHdG for long-distance bus drivers even after adjusted for smoking status. Exposure to chromate induces serum folate declining, which is associated with DNA damage and increased the level of urinary 8-OHdG [19]. Non-smoking male office workers have lower mean concentrations of urinary 8-OHdG (5.08 ± 2.56 μg/g creatinine) than people with occupational exposures to traffic exhaust or chromate. For non-smoking male office workers, environmental smoking and high level of CO_2_ were the major exposure sources at office. Therefore, it seems that the effect of environmental smoking exposure is less strong than that of traffic exhaust or chromate industry.

The levels of urinary 8-OHdG (3.17 µg/g creatinine) of female office workers in our study were lower than that of women exposed to wood smoke at home (ranges from 11.2–2270.0 µg/g creatinine) [36] or women working at Chinese restaurants (7.9 µg/g creatinine) [37]. In Taiwan, female office workers might cook at home at leisure time, however, they are not likely to be exposed to cooking fumes without ventilation at home.

In this study, there were limitations as well. This study did not measure nicotine in the office air. Instead, we measure the urinary cotinine, which is a major metabolite of nicotine specific to cigarette smoking, reflecting the exposure to tobacco smoking or environmental passive smoking. Besides, the smoking pack-year may not have association with urinary 8-OHdG in patients with lung cancer [23]. The level of urinary 8-OHdG reflects systemic DNA damage associated with exposure to various substances with oxidative stress effect. The office workers may expose to other pollutants in addition to tobacco smoke such as traffic exhaust and some diet contents inducing DNA damage. Using only cotinine as the marker might overestimate the association between tobacco smoking exposure and urinary 8-OHdG. However, compared with bus drivers or cooks, office workers exposure to these pollutants is limited. We also evaluated the association between urinary cotinine and 8-OHdG by controlling the indoor concentrations of carbon dioxide and volatile organic compounds. The significant correlation between urinary 8-OHdG, current smoking and urinary cotinine remained. In addition, a spot urine sample collected at the end of work may represent a short-time exposure to the office environment and the measured 8-OHdG may not infer the long-term exposure. Further study design is needed to estimate the dynamic changes on the level of 8-OHdG for a longer period to assess the total exposure at the end of work.

## 5. Conclusions

This study suggests that the level of urinary cotinine is an independent predictor of excess urinary 8-OHdG. Quitted smoking lowered urinary 8-OHdG concentrations. However, urinary 8-OHdG is also associated with indoor air quality using carbon dioxide as a marker. Efficient ventilation is needed to improve air quality in offices of high rise building.
